# Evaluation of Formalin-Fixed and FFPE Tissues for Spatially Resolved Metabolomics and Drug Distribution Studies

**DOI:** 10.3390/ph15111307

**Published:** 2022-10-23

**Authors:** Andreas Dannhorn, John G. Swales, Gregory Hamm, Nicole Strittmatter, Hiromi Kudo, Gareth Maglennon, Richard J. A. Goodwin, Zoltan Takats

**Affiliations:** 1 Department of Metabolism, Digestion and Reproduction, Imperial College London, London SW7 2AZ, UK; 2 Imaging & Data Analytics, Clinical Pharmacology and Safety Sciences, R&D, AstraZeneca, Cambridge CB4 0WG, UK; 3 Oncology Safety, Clinical Pharmacology and Safety Sciences, R&D, AstraZeneca, Cambridge CB4 0WG, UK; 4 Institute of Infection, Immunity and Inflammation, College of Medical, Veterinary and Life Sciences, University of Glasgow, Glasgow G12 8TA, UK

**Keywords:** mass spectrometry imaging, sample preparation, FFPE, DESI, MALDI

## Abstract

Fixation of samples is broadly used prior to the histological evaluation of tissue samples. Though recent reports demonstrated the ability to use fixed tissues for mass spectrometry imaging (MSI) based proteomics, glycomics and tumor classification studies, to date comprehensive evaluation of fixation-related effects for spatially resolved metabolomics and drug disposition studies is still missing. In this study we used matrix assisted laser desorption/ionization (MALDI) and desorption electrospray ionization (DESI) MSI to investigate the effect of formalin-fixation and formalin-fixation combined with paraffin embedding on the detectable metabolome including xenobiotics. Formalin fixation was found to cause significant washout of polar molecular species, including inorganic salts, amino acids, organic acids and carnitine species, oxidation of endogenous lipids and formation of reaction products between lipids and fixative ingredients. The slow fixation kinetics under ambient conditions resulted in increased lipid hydrolysis in the tissue core, correlating with the time-dependent progression of the fixation. Paraffin embedding resulted in subsequent partial removal of structural lipids resulting in the distortion of the elucidated biodistributions.

## 1. Introduction

Fresh frozen tissues are the current gold standard for metabolic MS imaging (MSI) applications. However, recent studies used fixed tissues such as formalin-fixed and formalin fixed paraffin embedded (FFPE) samples [1,2]. The main driving force here is to harmonize sample collection and usage for MSI applications with those for classical histology, for which latter commonly relies on FFPE tissues. Streamlined sample collection would reduce the associated workload and provide easy storage of formalin-fixed samples at room temperature, eliminating the need to store samples in a −80 °C or −150 °C freezer for long-term archival.

However, histological preparation protocols are optimized to improve the quality of optical images and inadvertently involve removal of endogenous metabolites. Recent reports highlight the suitability of FFPE tissues for MSI-based tissue classification [3], proteomics [4] and N-glycan [5] studies. The sensitivity of the latter ones is often limited in fresh-frozen tissue and tissue washes are commonly utilized to overcome ion suppression effects arising from the tissue metabolome and in particular from structural lipids [6,7]. Comparable increase in sensitivity were achieved through the preparation process for FFPE tissues, as the procedure removes small molecular species, in particular of lipids [8], from the tissue. This will ultimately alter analyte concentrations and potentially distort the detectable distributions. The reproducibility of the preparation and evaluation of the general effects on the tissue metabolome is often insufficiently evaluated in the existing reports.

As fixed tissues are generally considered to be stable, it is often neglected that the fixation in formalin is not an instantaneous step but occurs in a significant timeframe. Many enzymes show residual activity post-mortem for several hours or until formaldehyde cross-linking disrupts their activity. These enzymes include choline acetyltransferase (ChAT), glutamine synthetase (GS), glutamic acid decarboxylase (GAD), lactate dehydrogenase (LDH) and glycerol-3-phosphate dehydrogenase (GPDH), tyrosine- and dopamine-β-hydroxylase, monoamine oxidase (MAO) and phospholipases [9,10,11,12]. Residual enzyme activity in unfixed tissue compartments during the slow kinetics of the fixation could be a contributing factor to the established degradation of proteins and long DNA/RNA strands [13,14] and lipids [15,16] in FFPE tissues and could similarly impact metabolites of the rapidly changing tissue metabolome. Additionally, recent reports also indicate storage time dependent alterations of the tissue metabolome [17].

The reduced workload during sample collection and possibility to store samples at room temperature raised the question if samples collected in formalin are generally suitable for MSI based drug metabolism and pharmacokinetic (DMPK) and metabolomics studies. For the current study kidneys and livers of vehicle controls and rats cassette-dosed with terfenadine, diphenhydramine, dextromethorphan and losartan were divided upon sample collection and either snap-frozen or fixed in formalin until analysis respectively. Formalin-fixed liver specimens were additionally processed into a FFPE-multi-tissue block to evaluate the effects of downstream processing of the fixed tissues. The effects of the different collection and preparation procedures on the endogenous tissue metabolome and xenobiotics, including changes attributed to post-mortem enzyme activity, were evaluated by DESI- and MALDI-MSI.

## 2. Results

**Figure 1:**
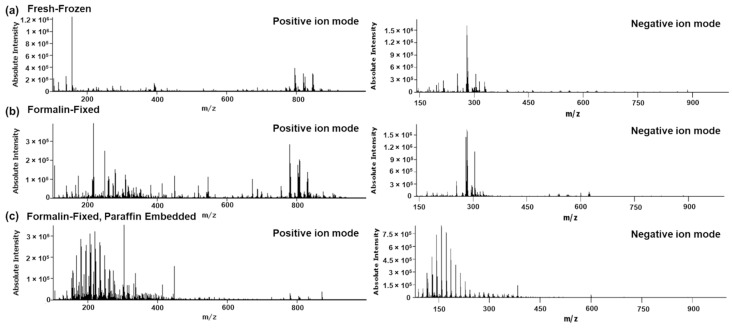
Representative mean spectra of (**a**) fresh-frozen, (**b**) formalin-fixed and (**c**) formalin-fixed, paraffin embedded rat liver specimens analyzed by DESI-MSI in positive and negative ion mode, respectively. All tissues were collected from the same animal.

Formalin-fixation and formalin-fixation combined with paraffin embedding of fresh frozen specimens inflicts significant alteration of the detectable tissue metabolome. Figure 1 shows representative mean spectra for the different treatment conditions of rat liver sections obtained from the same animal.

**Figure 2:**
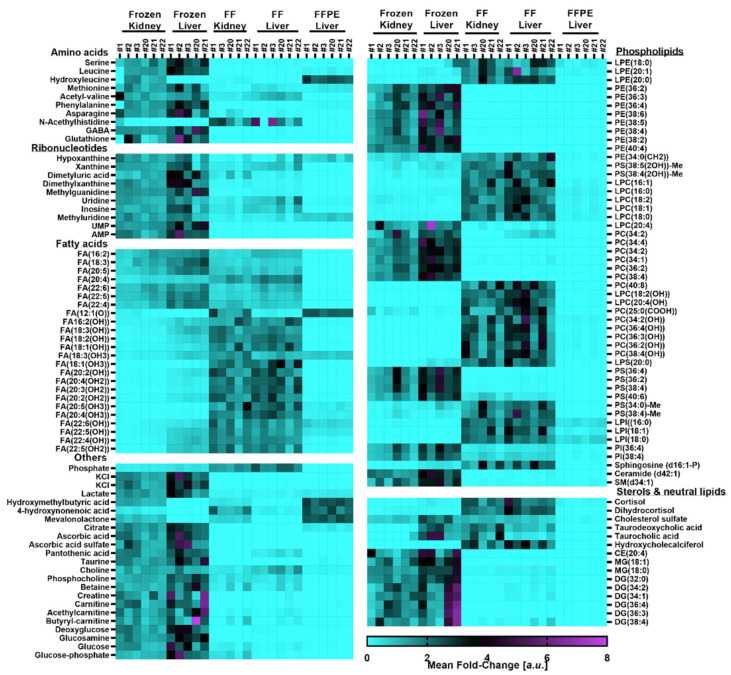
Mean-Fold changes of metabolite abundances as determined by DESI-MSI of fresh-frozen and formalin-fixed rat kidneys and fresh-frozen, formalin-fixed and formalin-fixed, paraffin embedded rat liver specimens respectively. Mean-fold changes were calculated to the average of all analyzed specimens in all treatment groups of one organ. The assigned numbers in the header refer to the individual animal IDs (i.e., #1 = Animal 1). Detailed information for the annotations can be found in Table S1. Detailed information regarding statistical significance for the changes can be found in Table S2.

Fresh-frozen tissue sections show a typical DESI spectrum with small molecules such as amino acids, organic amines, carbohydrates and carnitine species in the low mass range between *m*/*z* 100–500, lysophospholipids and diglycerides in the mass range between *m*/*z* 400–600 and glycerophospholipids such as phosphatidylcholines (PCs), phosphatidylethanolamines (PEs), phosphatidylserines (PSs), phosphatidylinositols (PIs), sterols and triglycerides in the mass range between *m*/*z* 600–1000. Whilst the spectrum of the formalin-fixed specimens is superficially comparable to the fresh frozen sample, the spectra are different with regard to both absolute and relative abundances of peaks observed, with the latter being particularly obvious in case of low mass range versus high mass range. The spectrum of the formalin-fixed, paraffin embedded sample is distinctively different with a prominent lack of signal in the lipid range and a broad envelope of signals in the mass range between *m*/*z* 100–500. The latter are generally associated with formaldehyde oligomers [18] which likely precipitated during the dehydration of the fixed tissues prior to embedding of the specimens in paraffin wax. Closer investigation revealed significant alterations of the endogenous tissue metabolome. The changes include washout of small molecular species such as amino acids, fatty acids and organic amines, oxidation of fatty acids (FAs) and lipids and reduced abundances of glycerophospholipids paired with increased abundances of lysophospholipids, indicative of lipid hydrolysis (Figure 2).

The washout of small molecular species was found to equally affect organic and inorganic species. The washout of potassium ions was most noticeable through the differences in the relative abundance of adduct ions. In agreement with literature reports [19], highly abundant potassium adducts of PCs, PEs, di- and triglycerides observed in fresh-frozen tissues were significantly reduced leaving sodium adducts as the main molecular ion species for these analyte classes in formalin-fixed samples. These observations are consistent with the lower abundance of KCl cluster in fixed samples (Figure 2). The lack in detection of analytes which primarily form potassium adducts might be misleading as it can be interpreted as absence of the analyte whilst it is likely that the predominant adduct ion is missing and the analyte of interest is detected as a different molecular ion species. FFPE tissues showed overall a significant removal of a wide range of analytes from small polar ones such as amino acids and organic amines to structural lipids. Class specific depletion of amine containing lipids upon formalin-fixation was previously reported [16] and has been linked to formylation of primary amine groups [15] present in lipid classes such as PEs and PSs. Tandem-MS based validation of the putative lipid annotations confirmed the presence of N-formylated PS species in formalin-fixed tissues (Figure S1a,b). In addition to formylation, methyl esterification of the carboxylic acid moiety was also observed. The methanol for the esterification originates from the commercially sourced formalin solution, where it is commonly added to prevent spontaneous polymerisation reactions [20,21] and oxidation of the formaldehyde. Additional modifications of the lipids include oxidation of unsaturated fatty acids in the lipids, explaining the lipid annotations in the heatmap in Figure 2. It is notable that the potassium adducts of lipids in positive ion mode are close in mass to sodium adducts of the corresponding oxidized lipid species, e.g., PC(36:2) as [M + K]^+^ has a theoretical mass of 824.557 whilst PC(36:2(OH)) as [M + Na]^+^ also has a theoretical mass of 824.577. As these species are too close in mass to be resolved by a quadrupole in a tandem-MS approach, the resulting overlapping MSMS spectra need to be carefully deconvoluted. Tandem-MS analysis of the corresponding ion in case of fresh-frozen sample resulted in a strong signal at *m*/*z* 162.955 which was reported to correspond to the headgroup fragment of a potassiated PC [22]. Formalin-fixed samples present the same fragment but additionally a fragment at *m*/*z* 146.981 which corresponds with the sodiated headgroup fragment (Figure S1c,d). With the exception of MS/MS spectra obtained from PIs, the MS/MS spectra acquired from formalin-fixed samples showed overall more complexity than the MS/MS spectra for the same masses obtained from fresh-frozen specimens (Supplementary Figure S1e). The significant alterations of the lipidome result in numerous additional peaks in the already complex lipid containing mass range between *m*/*z* 600–1000.

Pixel-wise Principal Component Analysis (PCA) performed on all samples found the largest variance in PC1, in both positive and negative ion mode, distinguishing FFPE tissues from fresh-frozen and formalin-fixed samples. Subsequent principal components were found to largely account for the effects of the formalin-fixation. Overall, even the difference between fresh-frozen liver and kidney specimens was lower than the variance introduced though the different sample treatments (Figure S2).

**Figure 3:**
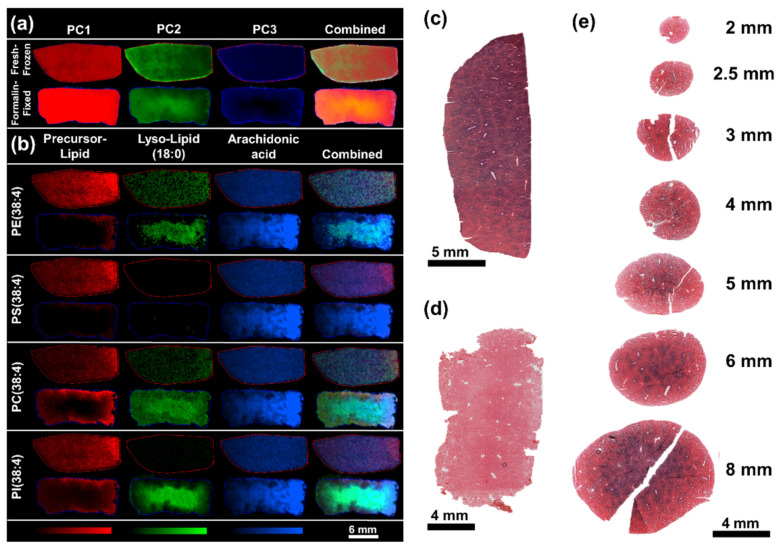
Comparison of representative fresh-frozen and formalin-fixed rat liver specimens. (**a**) Pixel-wise PCA of the tissue sections analyzed by DESI-MSI in negative ion mode. PC1 (red) contributed 16.65% PC2 (green) 9.38% and PC3 (blue) 5.52% of the total observed variance. (**b**) The regional differences found within the PC1–3 were found to correlate with the degradation of phospholipids in the tissue core and occurrence of the hydrolysis products lyso-lipids (18:0) and FA(20:4). (**c**) LDH stain of a frozen liver section compared to (**d**) LDH stain of a tissue section after fixation of the tissue for 24 h. (**e**) LDH stained tissue sections of rat liver punches in various sizes after 1 h of fixation.

The magnitude of many of the observed alterations, especially of lipid hydrolysis, oxidation and washout are likely to correlate with the storage duration of the specimens in formalin [23]. The prolonged storage in formalin might lead to an overestimation of the effect level compared to the results of fixing for histological evaluation, which are significantly shorter (24–72 h), but was still not found to induce unprecedented effects observed in specimens fixed for 24 h. This study was primarily designed to include effects of prolonged storage of specimens in formalin solution at room temperature to evaluate the effects observed after harmonized sample collection of tissues in formalin for both MSI and histological studies. As the specimens stored in formalin showed significant delocalization and washout of small molecular species making elucidation of an analyte’s origin within the tissue difficult to determine, specimens fixed for 24 h were used to evaluate effects of remnant enzyme activity within the tissues. Even after the shorter fixation, significant washout of low molecular weight analytes, especially around the tissue edges, made evaluation of standard tissue integrity benchmarks (e.g., glutamine/glutamate ratio) difficult to evaluate. However, pixel-wise PCA revealed regional differences with the abundance PC2 increasing from the tissue edge towards the tissue core and PC3 fading from the tissue edge towards the core (Figure 3a). The gradual changes were found to spatially correlate with the increasing abundance of lysophospholipids towards the tissue core and the predominance of intact lipids at the tissue edges. The gradual differences within the formalin-fixed specimens point towards hydrolysis of PEs, PCs and PIs into the corresponding lysophospholipids and free fatty acids, as seen for PE(38:4) (detected as [M − H]^−^ at *m*/*z* 766.540), PC(38:4) (detected as [M + Cl]^−^ at *m*/*z* 844.564), and PI(38:4) (detected as [M − H]^−^ at *m*/*z* 885.550) and the corresponding lysophospholipids LPE(18:0) (detected as [M − H]^−^ at *m*/*z* 480.310), LPC(18:0) (detected as [M + Cl]^−^ at *m*/*z* 558.334), and LPI(18:0) (detected as [M − H]^−^ at *m*/*z* 599.320) and free fatty acid FA(20:4), which is endogenously most likely to be arachidonic acid. Both, lysophospholipids and free fatty acid were predominantly found in the center core of the fixed specimens and less in the proximity to exposed edges and areas around larger blood vessels (Figure 3b).

The localization of the lipid fragments could be indicative of washout of the lipid fragments from the tissue edges or chemical hydrolysis, however, lower abundance of the originating phospholipids and limited degradation of PS(38:4) (detected as [M − H]^−^ at *m*/*z* 810.528) into LPS(18:0) (detected as [M − H]^−^ at *m*/*z* 524.299) point towards residual activity of phospholipases, in particular of phospholipase A2 (PLA_2_) which has been demonstrated to broadly accept PEs, PCs and PIs as substrates and to lesser extend PSs [24,25]. The increased hydrolysis of lipids in the tissue core can be explained through the diffusion and fixation kinetics of formaldehyde, which penetrates the tissue as the methanediol form and reacts relatively slowly as free aldehyde with amine and thiol groups within the tissue. Whilst fixed tissues are generally considered to be stable, the actual fixation is a slow process that can take several hours to completion (Figure S3). To demonstrate residual enzyme activity in fixed tissues and during the fixation, an aerobic lactate dehydrogenase stain (LDH stain) was performed. The LDH stain is an on-tissue enzyme activity stain which is widely used to determine cell viability in tissues, e.g., to determine the depth penetration of thermal injury to skin [26,27]. LDH is highly expressed throughout the liver and gives a strong stain in unfixed liver sections (Figure 3c). The enzymatic activity was fully arrested, even in relatively large rat liver specimen, after fixation for 24 h (Figure 3d). To recreate the conditions during the fixation process, which led to the degradation of lipids during the course of the 24 h fixation, liver punches of various sizes were collected from rat liver and fixed for 1 h. The LDH stain clearly demonstrates enzyme activity in the centre of all specimens with a diameter of 2.5 mm and above (Figure 3e). Analogue post-mortem alteration of the tissue metabolome through residual enzyme activity can be assumed for a variety of analytes in unfixed tissue compartments. These alterations might vary between tissues depending on the locally present enzymes. However, these changes might occur unnoticed when the affected metabolites are washed out of the tissue during the fixation or subsequent sample treatments. To preserve the integrity of the metabolic information contained in tissues, sample collection protocols involving fixing and subsequent MSI analysis should be appropriately planned. Ideally, the sample collection is performed using biopsy punches to ensure evenly sized specimens with comparable fixation effects. Even though the fixation kinetics might vary between tissue types, the maximum size of biopsies should ideally be kept under 2 mm to ensure swift and complete inactivation of metabolic enzymes throughout the specimens to ensure sufficient preservation of the tissue metabolome composition throughout the specimens. If analyte distributions can be elucidated from fixed tissues, post-mortem alterations should also be considered, and analyte distribution pattern should be thoroughly validated to avoid reporting of preparation artefacts.

Similar effects might arise from varying histological preparation protocols for the creation of FFPE tissue blocks. The protocols used might be adapted for different tissue types or vary between automated platforms and laboratories. Varying preparation protocols are a source of variability on the level of proteomic, genomic, transcriptomic and histological analysis of tissues [28] and have to be also considered as a source of variance at the metabolomic level. These variations are difficult to control or retrospectively account for, especially in archived samples where the metadata for the preparation might be missing. Furthermore, varying shapes, sizes and compositions of tissues introduce another source of variability which is even harder to control and even more difficult to standardize. FFPE-specific preparation artefacts can arise from the dehydration steps also removing large part of metabolic content. Incomplete removal of lipids or metabolites due to shape, size or different tissue composition of samples can significantly change the observed distribution patterns (Figure S4).

**Figure 4:**
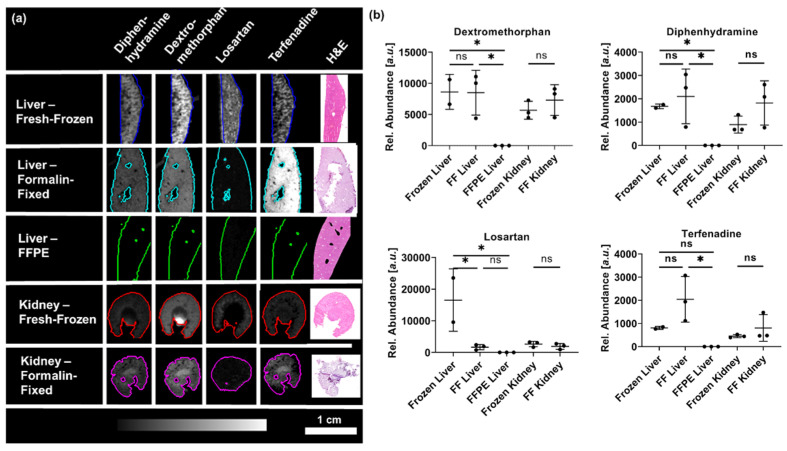
Effects of the sample collection and preparation on (**a**) representative distributions of the drugs and (**b**) mean abundance within the tissue sections for all biological replicates as determined by DESI-MSI. Statistical significance one-way ANOVA for liver samples, two-tailed *t*-test for kidneys. ns = *p* (>0.05), * = *p* (<0.05).

To evaluate the suitability of fixed samples for drug distribution and spatially resolved pharmacokinetic studies, the effects of the sample collection and processing were evaluated for the dosed drugs. The formalin-fixation did not significantly change the observed drug distributions and mean abundances of diphenhydramine, dextromethorphan or terfenadine in liver or kidney sections whilst losartan experienced significant washout from the liver specimens (Figure 4). Elucidation of drug distributions from FFPE samples was not possible as the drug abundances were close to background level.

Even though the mean abundances of the drugs were overall not significantly different, formalin-fixed samples showed higher variability between the biological replicates compared to the fresh-frozen samples. Whilst the mean abundances of diphenhydramine and terfenadine showed little variation between the fresh-frozen biological replicates, the mean drug abundances of the formalin-fixed specimens were more variable with samples showing up to 3-fold higher abundances compared to unfixed samples. The higher abundances might be rooted in removal of organic and inorganic salts from the tissues reducing ion suppression effects [6,29].

In addition to the altered abundances, distortion of the elucidated drug distributions could also be observed. The dosed drugs showed distinct zonation in the fresh frozen liver specimens, but the zonation of the drug distributions is lost within the formalin-fixed specimens. High resolution MSI data of fresh-frozen liver sections allowed to elucidate the distribution of terfenadine and its active metabolite fexofenadine in the liver. Terfenadine was detected in highest abundance in the periportal zone (hepatic zone 1), in contrast the endogenous lipid PC(36:2) which showed highest abundances in the centrilobular zone (hepatic zone 3) co-localizing with fexofenadine. Sections of the formalin-fixed counterpart were analyzed in the same experiments, but the dry tissue sections were largely destroyed during the data acquisition and no reasonable analyte distributions could be observed for these tissue sections.

**Figure 5:**
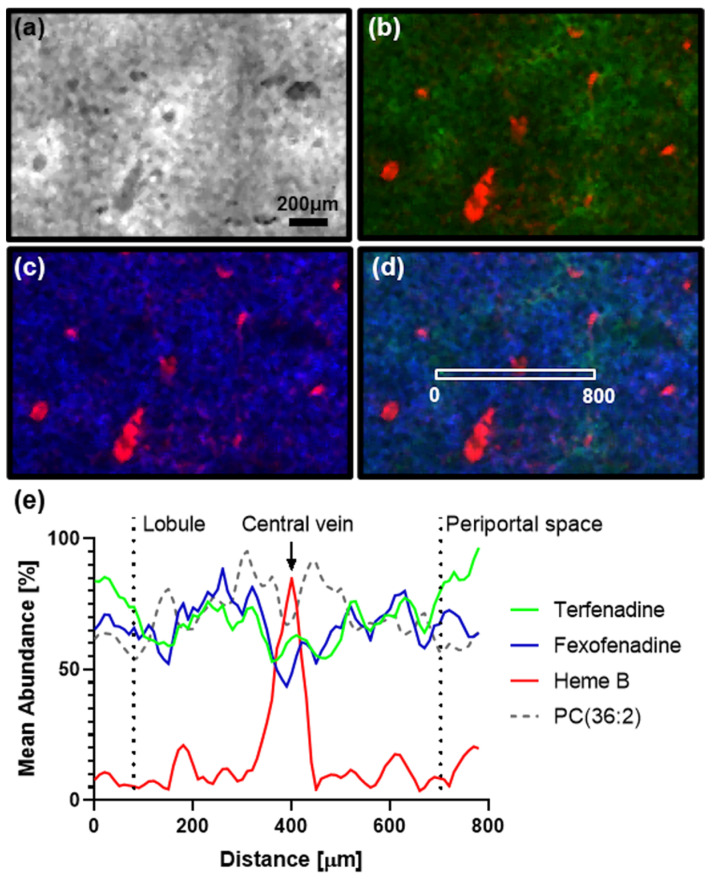
(**a**) Hepatic lobule of a fresh frozen liver section outlined by PC(36:2) (**b**) Terfenadine (green) in relative distribution to Heme B as determined by high resolution MALDI-MSI. (**c**) Distribution of Fexofenadine (active metabolite) compared to Heme B (**d**) RGB overlay of Terfenadine, Fexofenadine and Heme B. The box highlights the pixel used for the profile plot. (**e**) Profile plot of the normalized mean abundances of terfenadine, fexofenadine compared to PC(36:2) outlining the hepatic lobule and Heme B marking the central vein. Data is represented as mean of 3 separate lines, normalized to the highest pixel and cubically smoothed over 3 neighboring pixels to compensate for pixel-to-pixel variability.

Rapid freezing of the tissues at collection preserved the distribution of analytes within the specimens allowing accurate observation of rapidly changing analyte distributions (Figure 5). Formalin fixed samples did not preserve the endogenous analyte distributions resulting in loss and delocalization of analytes making retrospective elucidation of accurate biodistributions impossible.

**Figure 6:**
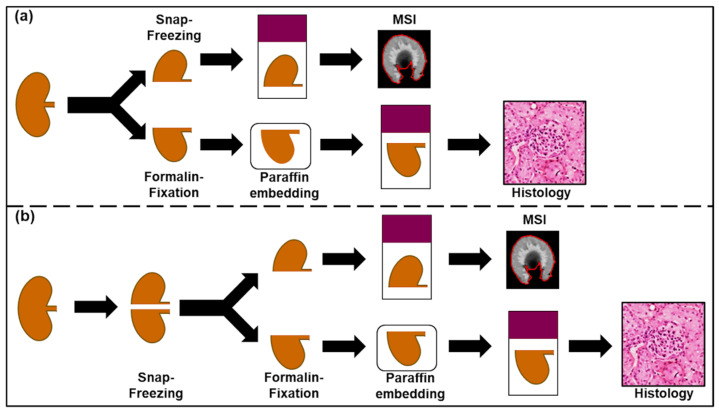
(**a**) Current sample collection workflow including separation of samples during collection. (**b**) facilitated workflow with snap freezing of all specimens during sample collection and subsequent fixation of specimens for histological evaluation.

Overall, the fresh-frozen samples were relatively easy to prepare with reasonable preservation of the tissue morphology, whilst formalin-fixed samples were very difficult to section as they were brittle. Most formalin-fixed tissue sections were fragile and had low adherence to the glass slide resulting in partial ablation during DESI and MALDI analysis. Most sections analyzed by MSI had no relevant remains suitable for histological evaluation. Even sections not analyzed by MSI were virtually impossible to H&E stain and most tissue sections were severely damaged or were removed from the slide during staining. Since the power of spatially resolved analysis lies in the ability to directly correlate the metabolic information with the underlying morphology, without the morphological information, interpretation of MSI data may at best inform about high-level changes of in the metabolome without enabling to link the information to the underlying cellular phenotypes. Paraffin embedding of formalin-fixed tissues was found to significantly improve tissue morphology, but at cost of significant removal of analytes from the tissues as described above. Taking advantage of the superior morphological preservation, the use of FFPE tissues for optical evaluation is well justified, however the use of such tissues for metabolomics and drug distribution studies remains questionable. To reduce workload during sample collection for prospective studies involving use of fresh-frozen and FFPE, we recommend snap freezing of all tissues during collection and subsequent branching of the tissue preparation workflows. As described above, snap freezing offers unparalleled preservation of endogenous biodistributions of analytes without compromising the tissue morphology when freezing and storage are done correctly [28,30]. Snap frozen samples can subsequently be processed into FFPE blocks through direct fixation of the frozen tissues in formalin and subsequent embedding in paraffin. Although this approach suffers the disadvantage that increases the numbers of samples requiring storage at ultra-low temperatures, it does benefit from the ability to decouple sample collection and processing (Figure 6).

Frozen samples can be stored at ultra-low temperatures without compromising quality [28], allowing to schedule processing of tissues when capacity is available rather than having to process the tissues as dictated by the sample collection and the subsequent timings of the treatment protocols. Liver specimens collected and stored in formalin showed substantial alteration of tissue morphology whilst FFPE tissues prepared from frozen specimens showed little processing artefacts (Figure S5). The observed decrease in the quality of tissue morphology is likely to arise from osmotic damage due to the prolonged storage of the specimens in formalin [21]. However, as the liver is a fairly homogenous organ these findings should not be considered as valid representation of all tissues and the suitability of the suggested sample processing workflow for tissues comprised of a more complex mixture of morphological features may require further validation.

## 3. Discussion

Existing reports outline the possibility to use archived tissues for tissue classification studies and the availability of large tissue archives builds a basis for creation of background databases for MSI guided histological determination of tumor margins. FFPE tissues are widely available in form of tissue micro arrays (TMAs), often containing 96 or more tissue cores. Analysis of slides prepared from TMAs allow for analysis to scale and could be a powerful tool in the generation of the underlying databases with sufficiently large sample numbers allowing accurate classification results. However, potential loss of metabolites, xenobiotics and lipids biases the biochemical information that can be obtained from fixed tissues. Their use to gain insights into the structure of molecular interaction networks responsible for tissue function in health and disease should be considered carefully. Selective loss and alteration of the tissue metabolome as well as potential introduction of artefacts during fixation and subsequent preparation and storage might also bias results. Overall, fresh frozen samples should be preferred as material for MSI based metabolomics and drug distribution studies to allow accurate elucidation of biodistributions. However, if prospective studies cannot source sufficient amounts of high quality fresh-frozen tissues, careful validation of preparation effects should be considered to limit the likelihood of drawing biased conclusions.

## 4. Materials and Methods

### 4.1. Chemicals

2,5-dihydroxybenzoic acid (DHB), 9-Aminiacridin hydrochloride (9-AA) trifluoroacetic acid (TFA), paraplast histology grade paraffin, terfenadine, dextromethorphan hydrobromide and diphenhydramine hydrochloride, nitrotetrazolium blue chloride, sodium chloride, 2 mM sodiumhydroxide solution, lactic acid, Nicotinamide adenine dinucleotide hydrate, polypep and Gly-Gly were purchased from Merck (Darmstadt, Germany). Methanol, water, iso-pentane, ethanol, xylene, isopropanol and acetonitrile (ACN) were obtained from Fisher Scientific (Waltham, MA, USA). Losartan-potassium salt was obtained from Cambridge Bioscience (Cambridge, UK). All solvents used were of analytical grade or higher.

### 4.2. Animals and Dosing

Experiments were performed using adult male Hans Wistar rats (approximate weight 260 g) obtained from Charles River Laboratories (Margate, Kent, U.K.). Prior to dosing the animals were acclimatized on site for a minimum of 3 days. The four compounds were co-administered by oral gavage at a dosing level of 25 mg/kg/drug. Terfenadine, losartan, diphenhydramine and dextromethorphan were formulated in 5% dimethyl sulfoxide/95% (30% *w*/*v* Captisol in water). Samples were collected 2 h after administration of a single dose. Vehicle controls were included in the study. Liver and kidneys of the animals were dissected and separated. One half of the left kidney and one liver lobe were collected and stored in 10% neutrally buffered formalin (Leica Biosystems, Nussloch, Germany). The remaining liver was immediately snap frozen in dry ice chilled isopentane. To prevent fracturing of the kidney specimens, kidneys were snap frozen in dry ice chilled isopropanol followed by a wash in dry ice chilled isopentane to wash off excess isopropanol. The frozen tissue samples were stored at −80 °C until further processing. All tissue dissection was performed by trained AstraZeneca staff (project license 40/3484, procedure number 10). To evaluate the effects of short-term fixation, vehicle dosed rat liver specimens were fixed in formalin for 24 h and immediately prepared as described below. A total of n = 6 fresh-frozen (3 dosed, 3 vehicle controls) and n = 5 formalin-fixed kidneys (3 dosed, 2 vehicle controls) and n = 5 frozen (2 dosed, 3 vehicle controls), n = 6 formalin-fixed (3 dosed, 3 vehicle controls), and n = 6 FFPE (3 dosed, 3 vehicle controls), respectively, were used to evaluate the effects of fixation and storage on the tissue metabolome and xenobiotics. Effects of 24 h formalin fixation were evaluated on n = 3 frozen and formalin-fixed liver specimens collected from the vehicle control group. Size dependent fixation effects were evaluated on tissue punches of different sizes collected from fresh-frozen control rat liver. To allow collection of uniformly sized punches, the tissue was allowed to warm up to −20 °C for 2 h and punches of different sizes (2, 2.5, 3, 4, 5, 6 and 8 mm) were collected. The tissue punches were fixed for 1 h in 4% formaldehyde before being snap-frozen in dry ice chilled isopentane. To facilitate sectioning of the samples, the frozen specimens were co-embedded in a HPMC/PVP-based hydrogel following a previously published protocol [31] forming multi-tissue blocks containing the different sized specimens. Effects of 1 h fixation of enzymatic activity were evaluated with n = 3 per punch size.

### 4.3. Tissue Preparation

To enable cryo-sectioning of the formalin-fixed samples, the specimens were gradually washed for 1 min in 70% ethanol followed by 1 min in 100% ethanol to remove the adherent fixation buffer and snap frozen in dry ice chilled iso-pentane. Liver specimens selected for formalin-fixation, paraffin embedding were dehydrated and cleansed as described in Table 1 and subsequently co-embedded in a multi-tissue FFPE block.

### 4.4. Tissue Sectioning

Sectioning of frozen specimens was performed on a CM3050 S cryostat (Leica Biosystems, Nussloch, Germany) at a section thickness of 10 µm. The specimens were mounted with frozen milli-Q water on the sample holder. The chamber temperature was set to −20 °C whilst the sample was held at −16 °C. For each animal, all matched tissue sections of formalin-fixed and fresh frozen tissues were mounted adjacent on one slide to achieve highest comparability of the data. Formalin-fixed, paraffin embedded tissues were sectioned to a thickness of 10 µm at room temperature on a microtome (Finesse ME+, Thermo Scientific, Waltham, MA, USA) and straightened on a water bath held at 40 °C. The sections were fixed onto the slides by backing them for 1 h at 63 °C. Prior to all MSI experiments the paraffin was removed by washing the slides for 1 min in xylene [32] followed by immediate drying under nitrogen. Samples were prepared on non-conductive SuperFrost microscope slides (Thermo Scientific, Waltham, MA, USA) for DESI experiments and hematoxylin and eosin (H&E) staining, whilst samples prepared for MALDI experiments were mounted onto conductive ITO slides (Bruker Daltonik, Bremen, Germany). Tissue sections for lactate dehydrogenase staining were mounted onto TOMO Adhesion Microscope Slides (Matsunami Glass Ind. Ltd., Japan).

### 4.5. DESI-MSI

Untargeted analysis was performed on a Q-Exactive plus mass spectrometer (Thermo Scientific, Bremen, Germany) due to the high mass resolving power of the mass analyzer. The instrument was equipped with an automated DESI ion source (Prosolia Inc., Zionsville, IN, USA). Data was acquired with a spatial resolution of 100 µm. The data was acquired in positive detection mode between *m*/*z* 100 to 1000 and between *m*/*z* 80–1000 in negative ion mode respectively. The nominal mass resolution was set to 70,000 and the injection time was fixed to 150 ms. The resulting scan rate was 3.8 pixel/s. The setup was used with a home-built DESI sprayer [33]. It was operated with a mixture of 95 % methanol, 5 % water. The DESI solvent was delivered with a flow rate of 1.5 µL/min and nebulized with nitrogen at a backpressure of 6 bar. The resulting .raw files were converted into .mzML files using ProteoWizard msConvert [34] (version 3.0.4043) before being compiled to .imzML files (imzML converter [35] version 1.3). All subsequent data processing was performed in SCiLS Lab (version 2019c, Bruker Daltonik, Bremen, Germany). All ion images are displayed normalized to the total ion current (TIC) and with a weak denoising filter applied to compensate for pixel-to-pixel variability of the data generating smoother images.

MS/MS experiments were performed with the same instrument parameters as listed above. The data were acquired by running line scans across the tissue and collecting data with an increasing collision energy to acquire a variety of fragment ions. Applicable spectra resulting in information rich MS/MS spectra were subsequently averaged and used for structure elucidation of the precursor ions. The injection time was fixed to 250 ms for all tandem-MS experiments.

### 4.6. MALDI-MSI

MALDI analysis was performed on a RapifleX Tissuetyper instrument (Bruker Daltonik, Bremen, Germany). DHB prepared in 50:50:0.1 ACN:water:TFA was used as MALDI matrix. An automated sprayer system (TM-Sprayer, HTX technologies, Chapel Hill, NC, USA) was used for spray deposition of the MALDI matrices following a previously reported protocol for DHB [36] MALDI experiments were performed with a spatial resolution of 10 µm. All MALDI experiments were performed in the mass range between *m*/*z* 100 and 1000 in positive ion mode. Final spectra were computed by summing up the spectra of 100 laser shots. The instrument was operated with a laser repetition rate of 10 kHz. The raw data was directly uploaded and processed in either FlexImaging (Bruker Daltonik, Bremen, Germany) or SCiLS lab (Version 2019c) software packages for in-depth processing. All MALDI data and images were TIC normalized to compensate for spectra-to-spectra variation of the data [37].

### 4.7. Statistical Analysis

Discriminating features differentiating the different treatments were identified in SCiLS lab using the receiver-operator-curve (ROC) function. For further investigation, features with a ROC-values above 0.75 were carried forward. Features presenting chemical background were manually identified based on the resulting ion images and removed from the feature list. The remaining featured were annotated using accurate mass matching against established databases such as METLIN (https://metlin.scripps.edu), human metabolome database (http://www.hmdb.ca) and LipidMaps (https://www.lipidmaps.org). The maximum allowed mass error was 6 ppm between the mean measured and the theoretical mass. Statistical significance for the annotated features was determined from all pixel of each treatment group and testing using the Kruskal–Wallis test followed by Dunn’s test for multiple comparisons performed in GraphPad Prism (V. 8.0.1) (GraphPad Software, San Diego, CA, USA).

### 4.8. Lactate Dehydrogenase Staining

Lactase dehydrogenase staining was performed following a previously published protocol [38]. Briefly, tissue sections were dried for approximately 1 h at room temperature. The staining area was surrounded with a hydrophobic pen and the slides were rinsed in PBS for two times 5 min. The reaction solution was applied (polypep base solution (5% polypep, 0.75% NaCl, 2 mM Gly-Gly, adjusted to pH 8) containing 1.75 mg/mL NAD, 3 mg/mL nitrotetrazolium blue chloride and 60 mM lactic acid) and the slides incubated at 37 °C for 3–4 h. The slides were rinsed for 30 s in hot water followed by PBS. The slides were briefly dipped for 1 s in eosin solution to counterstain the tissues, washed in PBS, acetone, acetone/xylene, xylene before being coverslipped. Negative controls without addition of NAD were carried alongside all LDH stains.

## 5. Conclusions

High quality fresh frozen tissues are most widely used to probe tissue biology using MSI-based techniques as these tissues closely resemble the in vivo situation. While fixed material is readily available, its use for such purposes must be critically considered. The here presented work clearly highlights the potential of processing artefacts in fixed tissues that can skew data acquisition and interpretation. Furthermore, postmortem remnant enzyme activity can alter the tissue metabolome even during the fixation of larger specimens. When designing studies collecting tissue specimens in formalin for subsequent MSI analysis, the specimens should ideally be collected with a biopsy punch of 2.5 mm or less to allow swift and complete fixation of the entire biopsy. Although such sampling reduces potential artefacts arising from enzyme activity, it cannot overcome the observed washout and delocalization of small molecular species. The effects determined for the dosed drugs highlights the importance to use high-quality fresh frozen material for DMPK purposes. Quantitative determination of drug disposition is virtually impossible from fixed tissues as the preparation artefacts are non-reproducible. Reducing the duration in which tissues are immersed in fixatives and solvents might limit such effects onto the tissue metabolome. However, drastic deviation from times specified in established protocols might be even worse as partly fixed tissues will have compromised stability and might undergo increased degradation during storage. To validate accurate elucidation of metabolite distributions from fixed tissues at least a sub-set of the study should be compared to fresh frozen material to ensure that conclusions are accurate and not biased by the sample preparation. Careful consideration and validation of features detected by MSI against established approaches such as LC-MS analysis or nuclear magnetic resonance spectroscopy can greatly help to increase the certainty of metabolite annotations, especially if they are obtained from FFPE material which can show large polymer cluster, especially in the low mass range.

If both, fresh frozen and fixed tissues are required for a given study, the use of the facilitated workflow described above can help reduce workload at the point of sample collection and free up resources by decoupling the sample collection and fixation.

## Figures and Tables

**Table 1:** Preparation procedure for the paraffin embedding of formalin-fixed samples.

Treatment	Duration [h]	Temperature [°C]
10% Formalin	1	40
70% EtOH	1	40
EtOH	1	40
EtOH	1	40
Xylene	0.5	40
Xylene	0.5	40
Paraffin	1	63

## Data Availability

Data is contained within the article and supplementary material.

## Supplementary Materials

The following supporting information can be downloaded at: https://www.mdpi.com/article/10.3390/ph15111307/s1, Table S1: Annotation details for the heatmap features; Table S2: Statistical information for annotated heatmap features; Figure S1: MS/MS spectra of heatmap lipids; Figure S2: Pixel-wise PCA performed on sections from all tissue sections; Figure S3: Progression of the fixation front in rat over time; Figure S4: Distribution of PI(38:4) in fresh-frozen, formalin-fixed and formalin-fixed, paraffin embedded rat liver; Figure S5: H&E stained FFPE sections prepared from tissue collected and stored in 10% formalin or snap-frozen upon collection and subsequently fixed in 10% formalin.
