# Supplementary material for: Evaluation of Formalin-Fixed and FFPE Tissues for Spatially Resolved Metabolomics and Drug Distribution Studies
Source: Pharmaceuticals (Basel). 2022 Oct 23;15(11):1307. doi: 10.3390/ph15111307 (PMC9697942; doi:10.3390/ph15111307)
Supplement: Supplementary file 1 [file pharmaceuticals-15-01307-s001.zip › pharmaceuticals-1939014-supplementary.pdf]

Supplementary Information “Evaluation of formalin-fixed and FFPE tissues for spatially resolved metabolomics and drug distribution studies”

Andreas Dannhorn[1,2], John Swales[2], Gregory Hamm[2], Nicole Strittmatter[2], Hiromi Kudo[1], Gareth Maglennon[3], Richard JA Goodwin[2,4], Zoltan Takats[1,*]

1) Department of Metabolism, Digestion and Reproduction, Imperial College London, London, UK

2) Imaging & Data Analytics, Clinical Pharmacology and Safety Sciences, R&D, AstraZeneca, Cambridge, UK

3) Oncology Safety, Clinical Pharmacology and Safety Sciences, R&D, AstraZeneca, Cambridge, UK

4) Institute of Infection, Immunity and Inflammation, College of Medical, Veterinary and Life Sciences, University of Glasgow, Glasgow, UK

Table S1: Annotation details for the heatmap features:

**Annotation**	**Adduct**	**Measured *m/z***	**Theoretical *m/z***	**Error [ppm]**	**Molecular formula**	**MS/MS**
Serine	[M-H2O-H]-	86.024	86.025	5	$C_3H_7NO_3$	
Leucine	[M+Cl]-	166.064	166.064	0	$C_6H_{13}NO_2$	
Hydroxyleucine	[M-H]-	146.082	146.082	0	$C_6H_{13}NO_3$	
Methionine	[M-H]-	148.044	148.044	1	$C_5H_{11}NO_2S$	
Acetyl-valine	[M-H]-	158.083	158.082	4	$C_7H_{13}NO_3$	
Phenylalanine	[M-H]-	164.072	164.072	0	$C_9H_{11}NO_2$	
Asparagine	[M+Cl]-	167.024	167.023	4	$C_4H_8N_2O_3$	
N-Acethylhistidine	[M+Na]+	220.070	220.069	2	$C_8H_{11}N_3O_3$	
GABA	[M-H]-	102.056	102.056	2	$C_4H_9NO_2$	
Glutathione	[M-H]-	306.077	306.077	0	$C_{10}H_{17}N_3O_6S$	
Hypoxanthine	[M+K]+	175.002	175.002	1	$C_5H_4N_4O$	
Xanthine	[M-H]-	151.026	151.026	2	$C_5H_4N_4O_2$	
Dimetyluric acid	[M-H]-	195.052	195.052	0	$C_7H_8N_4O_3$	
Dimethylxanthine	[M+Cl]-	215.034	215.034	0	$C_7H_8N_4O_2$	
Methylguanidine	[M-H2O-H]-	146.047	146.047	1	$C_6H_7N_5O$	
Uridine	[M-H]-	243.063	243.062	3	$C_9H_{12}N_2O_6$	
Inosine	[M-H]-	267.072	267.073	5	$C_{10}H_{12}N_4O_5$	
Methyluridine	[M+Cl]-	293.055	293.055	0	$C_{10}H_{14}N_2O_6$	
UMP	[M-H]-	323.029	323.029	2	$C_9H_{13}N_2O_9P$	
AMP	[M-H]-	346.056	346.056	0	$C_{10}H_{14}N_5O_7P$	
FA(16:2)	[M-H]-	251.203	251.202	5	$C_{16}H_{28}O_2$	
FA(18:3)	[M-H]-	277.216	277.217	5	$C_{18}H_{30}O_2$	
FA(20:5)	[M-H]-	301.217	301.217	0	$C_{20}H_{30}O_2$	
FA(20:4)	[M-H]-	303.233	303.233	0	$C_{20}H_{32}O_2$	
FA(22:6)	[M-H]-	327.235	327.233	5	$C_{22}H_{32}O_2$	
FA(22:5)	[M-H]-	329.250	329.249	5	$C_{22}H_{34}O_2$	
FA(22:4)	[M-H]-	331.266	331.264	4	$C_{22}H_{36}O_2$	
FA(12:1(O))	[M+Na]+	235.130	235.130	1	$C_{12}H_{20}O_3$	
FA16:2(OH))	[M-H]-	267.196	267.197	2	$C_{16}H_{28}O_3$	
FA(18:3(OH))	[M-H]-	293.213	293.212	3	$C_{18}H_{30}O_3$	
FA(18:2(OH))	[M-H]-	295.229	295.228	3	$C_{18}H_{32}O_3$	
FA(18:1(OH))	[M-H]-	297.244	297.244	3	$C_{18}H_{34}O_3$	
FA(18:3(OH3)	[M-H]-	325.203	325.202	4	$C_{18}H_{30}O_5$	
FA(18:1(OH3))	[M-H]-	329.235	329.233	4	$C_{18}H_{34}O_5$	
FA(20:2(OH))	[M-H]-	323.258	323.259	5	$C_{20}H_{36}O_3$	
FA(20:4(OH2))	[M-H]-	335.223	335.223	2	$C_{20}H_{32}O_4$	
FA(20:3(OH2))	[M-H]-	337.239	337.238	2	$C_{20}H_{34}O_4$	
FA(20:2(OH2))	[M-H]-	339.255	339.254	2	$C_{20}H_{36}O_4$	
FA(20:5(OH3))	[M+Na]+	373.197	373.198	2	$C_{20}H_{30}O_5$	
FA(20:4(OH3))	[M+Na]+	375.213	375.214	1	$C_{20}H_{32}O_5$	
FA(22:6(OH))	[M-H]-	343.228	343.228	0	$C_{22}H_{32}O_3$	
FA(22:5(OH))	[M-H]-	345.243	345.244	0	$C_{22}H_{34}O_3$	
FA(22:4(OH))	[M-H]-	347.259	347.259	0	$C_{22}H_{36}O_3$	
FA(22:5(OH2))	[M-H]-	361.241	361.238	6	$C_{22}H_{34}O_4$	
Phosphate	[M-H]-	96.970	96.970	3	H_3PO_4	
KCl	[M+Cl]-	108.902	108.903	5	KCl	
KCl	[M+K]+	112.896	112.895	5	KCl	
Lactate	[M-H]-	89.025	89.024	2	$C_3H_6O_3$	
Hydroxymethylbutyric acid	[M-H]-	117.055	117.056	4	$C_5H_{10}O_3$	
Hydroxynonenoic acid	[M+Na]+	195.100	195.099	5	$C_9H_{16}O_3$	
Mevalonolactone	[M-H]-	129.056	129.056	2	$C_6H_{10}O_3$	
Citrate	[M-H2O-H]-	173.008	173.009	4	$C_6H_8O_7$	
Ascorbic acid	[M-H]-	175.024	175.025	5	$C_6H_8O_6$	
Ascorbic acid sulfate	[M-H]-	254.983	254.982	3	$C_6H_8O_9S$	
Pantothenic acid	[M-H]-	218.102	218.103	6	$C_9H_{17}NO_5$	
Taurine	[M-H]-	124.007	124.007	1	$C_2H_7NO_3S$	
Choline	[M+H]+	104.107	104.107	3	$C_5H_{13}NO$	
Phosphocholine	[M+H]+	184.074	184.073	2	$C_5H_{14}NO_4P$	
Betaine	[M+H]+	118.087	118.086	4	$C_5H_{11}NO_2$	
Creatine	[M+H]+	132.077	132.077	1	$C_4H_9N_3O_2$	
Carnitine	[M+K]+	200.069	200.068	1	$C_7H_{15}NO_3$	
Acethylcarnitine	[M+H]+	204.123	204.123	0	$C_9H_{17}NO_4$	
Butyryl-carnitine	[M+H]+	232.154	232.154	2	$C_{11}H_{21}NO_4$	
Deoxyglucose	[M+Cl]-	199.037	199.038	4	$C_6H_{12}O_5$	
Glucosamine	[M+Cl]-	214.048	214.049	5	$C_6H_{13}NO_5$	
Glucose	[M+K]+	219.026	219.027	2	$C_6H_{12}O_6$	

Glucose-phosphate	[M-H]-	259.021	259.022	4	$C_6H_{13}O_9P$	
LPE(18:0)	[M+Na]+	504.305	504.306	1	$C_{23}H_{48}NO_7P$	
LPE(20:1)	[M-H]-	506.325	506.325	1	$C_{25}H_{60}NO_7P$	
LPE(20:0)	[M-H]-	508.340	508.341	1	$C_{25}H_{62}NO_7P$	
PE(36:2)	[M+K]+	782.508	782.509	1	$C_{41}H_{78}NO_8P$	Yes
PE(36:3)	[M-H]-	740.525	740.524	2	$C_{41}H_{76}NO_8P$	Yes
PE(36:4)	[M+K]+	778.476	778.478	2	$C_{41}H_{74}NO_8P$	Yes
PE(38:6)	[M-H]-	762.511	762.508	4	$C_{43}H_{74}NO_8P$	Yes
PE(38:5)	[M-H]-	764.526	764.524	4	$C_{43}H_{76}NO_8P$	Yes
PE(38:4)	[M-H]-	766.542	766.539	3	$C_{43}H_{78}NO_8P$	Yes
PE(38:2)	[M+K]+	810.539	810.540	2	$C_{43}H_{82}NO_8P$	
PE(40:4)	[M+K]+	834.539	834.540	2	$C_{45}H_{82}NO_8P$	
PE(34:0(CH2))	[M+Na]+	754.533	754.535	2	$C_{40}H_{78}NO_8P$	
PE(44:10(OH))	[M-H]-	854.536	854.534	2	$C_{49}H_{78}NO_9P$	
PE(44:9(OH))	[M-H]-	856.551	856.550	2	$C_{49}H_{80}NO_9P$	
LPC(16:1)	[M+Na]+	516.305	516.306	1	$C_{24}H_{48}NO_7P$	
LPC(16:0)	[M+Na]+	518.320	518.321	3	$C_{24}H_{50}NO_7P$	
LPC(18:2)	[M+Na]+	542.320	542.321	3	$C_{26}H_{50}NO_7P$	
LPC(18:1)	[M+Na]+	544.336	544.337	2	$C_{26}H_{52}NO_7P$	
LPC(18:0)	[M+Na]+	546.352	546.352	1	$C_{26}H_{54}NO_7P$	
LPC(20:4)	[M+K]+	582.294	582.295	1	$C_{28}H_{50}NO_7P$	
PC(34:2)	[M+H]+	758.568	758.569	1	$C_{42}H_{80}NO_8P$	Yes
PC(34:4)	[M+K]+	792.491	792.493	3	$C_{42}H_{76}NO_8P$	Yes
PC(34:2)	[M+K]+	796.523	796.525	2	$C_{42}H_{80}NO_8P$	Yes
PC(34:1)	[M+K]+	798.538	798.540	3	$C_{42}H_{82}NO_8P$	Yes
PC(36:2)	[M+K]+	824.554	824.557	3	$C_{44}H_{84}NO_8P$	Yes
PC(38:4)	[M+K]+	848.555	848.556	1	$C_{46}H_{84}NO_8P$	Yes
PC(40:8)	[M+K]+	868.527	868.525	3	$C_{48}H_{80}NO_8P$	Yes
LPC(18:2(OH))	[M+Na]+	558.315	558.316	3	$C_{26}H_{50}NO_8P$	
LPC(20:4(OH)	[M+Na]+	582.314	582.316	3	$C_{28}H_{50}NO_8P$	
PC(25:0(COOH))	[M+Na]+	688.415	688.415	1	$C_{33}H_{64}NO_{10}P$	
PC(34:2(OH))	[M+Na]+	796.544	796.546	3	$C_{42}H_{80}NO_9P$	Yes
PC(36:4(OH))	[M+Na]+	820.544	820.546	2	$C_{44}H_{80}NO_9P$	Yes
PC(36:3(OH))	[M+Na]+	822.560	822.561	2	$C_{44}H_{82}NO_9P$	Yes
PC(36:2(OH))	[M+Na]+	824.575	824.577	3	$C_{44}H_{84}NO_9P$	Yes
PC(38:4(OH))	[M+Na]+	848.576	848.577	1	$C_{46}H_{84}NO_9P$	
LPS(20:0)	[M-H]-	552.332	552.331	1	$C_{26}H_{52}NO_9P$	
PS(36:4)	[M-H]-	782.500	782.498	3	$C_{42}H_{74}NO_{10}P$	Yes
PS(36:2)	[M-H]-	786.532	786.529	3	$C_{42}H_{76}NO_{10}P$	Yes
PS(38:4)	[M-H]-	810.533	810.529	5	$C_{44}H_{78}NO_{10}P$	Yes
PS(40:6)	[M-H]-	834.531	834.529	2	$C_{46}H_{78}NO_{10}P$	Yes
PS(34:0)-Me	[M-H]-	774.531	774.529	2	$C_{41}H_{78}NO_{10}P$	Yes
PS(38:4)-Me	[M-H]-	822.534	822.529	5	$C_{45}H_{78}NO_{10}P$	Yes
LPI((16:0) [M-H]$^-$	[M-H]-	571.288	571.289	1	$C_{25}H_{49}O_{12}P$	
LPI(18:1) [M-H]$^-$	[M-H]-	597.305	597.305	1	$C_{27}H_{51}O_{12}P$	
LPI(18:0) [M-H]$^-$	[M-H]-	599.321	599.320	1	$C_{27}H_{53}O_{12}P$	
PI(36:4) [M-H]$^-$	[M-H]-	857.522	857.519	4	$C_{45}H_{79}O_{13}P$	Yes
PI(38:4) [M-H]$^-$	[M-H]-	885.555	885.550	6	$C_{47}H_{83}O_{13}P$	Yes
Sphingosine (d16:1-P)	[M-H2O-H]-	332.198	332.200	5	$C_{16}H_{34}NO_5P$	
Ceramide (d42:1)	[M+K]+	688.599	688.600	2	$C_{42}H_{83}NO_3$	
SM(d34:1)	[M+K]+	741.529	741.532	4	$C_{39}H_{79}N_2O_6P$	
Cortisol	[M+Cl]-	397.177	397.179	5	$C_{21}H_{30}O_5$	
Dihydrocortisol	[M+Cl]-	399.193	399.194	5	$C_{21}H_{32}O_5$	
Cholesterol sulfate	[M-H]-	465.305	465.304	1	$C_{27}H_{46}O_4S$	
Taurodeoxycholic acid	[M-H]-	498.290	498.289	0	$C_{26}H_{45}NO_6S$	
Taurocholic acid	[M-H]-	514.283	514.284	3	$C_{26}H_{45}NO_7S$	
Hydroxycholecalciferol	[M+Na]+	423.322	423.323	3	$C_{27}H_{44}O_2$	
CE(20:4)	[M+K]+	711.545	711.547	3	$C_{47}H_{76}O_2$	
MG(18:1)	[M+K]+	395.255	395.255	0	$C_{21}H_{40}O_4$	
MG(18:0)	[M+K]+	397.270	397.271	2	$C_{21}H_{42}O_4$	
DG(32:0)	[M+K]+	607.472	607.469	4	$C_{35}H_{68}O_5$	
DG(34:2)	[M+K]+	631.468	631.469	3	$C_{37}H_{68}O_5$	
DG(34:1)	[M+K]+	633.485	633.485	0	$C_{37}H_{70}O_5$	
DG(36:4)	[M+K]+	655.467	655.469	3	$C_{39}H_{68}O_5$	
DG(36:3)	[M+K]+	657.484	657.485	2	$C_{39}H_{70}O_5$	
DG(38:4)	[M+K]+	683.499	683.501	2	$C_{41}H_{72}O_5$	

Table S2: Statistical information for annotated heatmap features:

**Annotation**	**Polarity**	**Median Abundances [*a.u.*]***					**Statistical significance P****			
		**Fresh-Frozen Kidney**	**Formalin-Fixed Kidney**	**Fresh-Frozen Liver**	**Formalin-Fixed Liver**	**FFPE liver**	**Fresh-Frozen v Formalin-Fixed Kidney**	**Fresh-Frozen v Formalin-Fixed Liver**	**Fresh-Frozen v FFPE Liver**	**Formalin-Fixed v FFPE liver**
Serine	-	2968	0.000	1445	0.000	0.000	<0.001	<0.001	<0.001	<0.001
Leucine	-	2192	0.000	5368	0.000	0.000	<0.001	<0.001	<0.001	<0.001
Hydroxy-leucine	-	4940	0.000	0.000	0.000	18802	<0.001	<0.001	<0.001	<0.001
Methionine	-	4382	1697	0.000	2938	0.000	<0.001	<0.001	<0.001	<0.001
Acetyl-valine	-	5350	1974	1741	2938	71950	<0.001	<0.001	<0.001	<0.001
Phenylalanine	-	2689	0.000	2528	0.000	0.000	<0.001	<0.001	<0.001	<0.001
Asparagine	-	8127	874.0	20272	0.000	1591	<0.001	<0.001	<0.001	<0.001
Acethyl-histidine	+	0.000	3347	0.000	1603	0.000	<0.001	<0.001	<0.001	<0.001
GABA	-	40195	0.000	3147	0.000	0.000	<0.001	<0.001	<0.001	<0.001
Glutathione	-	14495	0.000	33063	0.000	0.000	<0.001	<0.001	<0.001	ns
Hypoxanthine	+	5631	0.000	1447	0.000	0.000	<0.001	<0.001	<0.001	<0.001
Xanthine	-	86253	20129	42701	2938	0.000	<0.001	<0.001	<0.001	<0.001
Dimetyluric acid	-	38836	18278	232805	0.000	0.000	<0.001	<0.001	<0.001	<0.001
Dimethylxanthine	-	337022	26728	501221	0.000	0.000	<0.001	<0.001	<0.001	<0.001
Methyl-guanidine	-	427032	2636	27990	1741	2093	<0.001	<0.001	<0.001	<0.001
Uridine	-	15874	3755	5165	0.000	0.000	<0.001	<0.001	<0.001	<0.001
Inosine	-	33698	12791	13512	5736	0.000	<0.001	<0.001	<0.001	<0.001
Methyluridine	-	774.4	834.0	5075	1057	2463	<0.001	<0.001	<0.001	<0.001
UMP	-	8795	0.000	10529	0.000	0.000	<0.001	<0.001	<0.001	ns
AMP	-	59441	0.000	14343	0.000	0.000	<0.001	<0.001	<0.001	ns
FA(16:2)	-	7151	4578	6194	3894	0.000	<0.001	<0.001	<0.001	<0.001
FA(18:3)	-	45320	42495	162445	66291	0.000	<0.001	<0.001	<0.001	<0.001
FA(20:5)	-	29111	34088	109682	44199	0.000	<0.001	<0.001	<0.001	<0.001
FA(20:4)	-	422356	1000000	458781	1245220	2480	<0.001	<0.001	<0.001	<0.001
FA(22:6)	-	109054	65832	188029	139123	0.000	<0.001	<0.001	<0.001	<0.001
FA(22:5)	-	47151	27765	131519	54772	0.000	<0.001	<0.001	<0.001	<0.001
FA(22:4)	-	27475	37325	80100	31703	0.000	<0.001	<0.001	<0.001	<0.001
FA(12:1(O))	+	0.000	7757	0.000	18471	281386	<0.001	<0.001	<0.001	<0.001
FA16:2(OH))	-	1575	12791	6185	5736	1056	<0.001	<0.001	<0.001	<0.001
FA(18:3(OH))	-	9043	197702	116059	206160	0.000	<0.001	<0.001	<0.001	<0.001
FA(18:2(OH))	-	11335	283032	87898	309557	1798	<0.001	<0.001	<0.001	<0.001
FA(18:1(OH))	-	8015	174157	26568	162739	2370	<0.001	<0.001	<0.001	<0.001
FA(18:3(OH3)	-	1270	10004	7600	10763	9325	<0.001	<0.001	<0.001	<0.001
FA(18:1(OH3))	-	2738	54362	1776	35624	2481	<0.001	<0.001	0.0339	<0.001
FA(20:2(OH))	-	0.000	8612	2299	12265	0.000	<0.001	<0.001	<0.001	<0.001
FA(20:4(OH2))	-	2554	67268	6589	63020	0.000	<0.001	<0.001	<0.001	<0.001
FA(20:3(OH2))	-	0.000	44502	3647	43371	1978	<0.001	<0.001	<0.001	<0.001
FA(20:2(OH2))	-	0.000	12600	2319	11590	0.000	<0.001	<0.001	<0.001	<0.001
FA(20:5(OH3))	+	0.000	5263	0.000	5122	977.9	<0.001	<0.001	<0.001	<0.001
FA(20:4(OH3))	+	0.000	12986	0.000	13915	1824	<0.001	<0.001	<0.001	<0.001
FA(22:6(OH))	-	1989	16678	9480	22441	9165	<0.001	<0.001	ns	<0.001
FA(22:5(OH))	-	798.8	7505	6577	10046	2270	<0.001	<0.001	<0.001	<0.001
FA(22:4(OH))	-	0.000	4272	2519	4190	0.000	<0.001	<0.001	<0.001	<0.001
FA(22:5(OH2))	-	0.000	7833	3964	8265	0.000	<0.001	<0.001	<0.001	<0.001
Phosphate	-	132675	318868	48681	111410	3935	<0.001	<0.001	<0.001	<0.001
KCL	-	10452	0.000	4815	0.000	0.000	<0.001	<0.001	<0.001	ns
KCl	+	8183	0.000	6827	0.000	0.000	<0.001	<0.001	<0.001	ns
Lactate	-	693776	67077	1445	0.000	101149	<0.001	<0.001	<0.001	<0.001
Hydroxymethyl butyric acid	-	4450	997.3	1549	9037	198339	<0.001	<0.001	<0.001	<0.001
Hydroxy-nonenoic acid	+	38836	18278	232805	0.000	0.000	<0.001	<0.001	<0.001	<0.001
Mevalono-lactone	-	8838	5717	6452	601.1	454411	<0.001	<0.001	<0.001	<0.001
Citrate	-	38616	0.000	24215	0.000	2633	<0.001	<0.001	<0.001	<0.001

Ascorbic acid	-	279919	0.000	181887	0.000	0.000	<0.001	<0.001	<0.001	<0.001
Ascorbic acid sulfate	-	22250	0.000	13496	366720	0.000	<0.001	<0.001	<0.001	<0.001
Pantothenic acid	-	87613	1536	4556	0.000	0.000	<0.001	<0.001	<0.001	<0.001
Taurine	-	337618	849.1	24886	601.1	1018	<0.001	<0.001	<0.001	<0.001
Choline	+	761457	230829	259560	239633	37457	<0.001	<0.001	<0.001	<0.001
Phospho-choline	+	5767	2293	3939	0.000	0.000	<0.001	<0.001	<0.001	<0.001
Betaine	+	95027	34383	162982	18829	0.000	<0.001	<0.001	<0.001	<0.001
Creatine	+	4120	0.000	0.000	0.000	0.000	<0.001	<0.001	<0.001	ns
Carnitine	+	12371	0.000	5185	0.000	0.000	<0.001	<0.001	<0.001	<0.001
Acethyl-carnitine	+	28411	1964	26494	0.000	0.000	<0.001	<0.001	<0.001	<0.001
Butyryl-carnitine	+	4274	0.000	4620	0.000	0.000	<0.001	<0.001	<0.001	<0.001
Deoxyglucose	-	38836	0.000	1833	0.000	0.000	<0.001	<0.001	<0.001	<0.001
Glucosamine	-	39800	521.0	85146	0.000	0.000	<0.001	<0.001	<0.001	<0.001
Glucose	-	18691	3195	45633	1858	795.3	<0.001	<0.001	<0.001	<0.001
Glucose-phosphate	-	22250	1201	12719	366720	0.000	<0.001	<0.001	<0.001	<0.001
LPE(18:0)	+	0.000	4565	0.000	0.000	0.000	<0.001	<0.001	ns	<0.001
LPE(20:1)	-	0.000	2036	0.000	2485	0.000	<0.001	<0.001	ns	<0.001
LPE(20:0)	-	0.000	10506	0.000	1116	0.000	<0.001	<0.001	<0.001	<0.001
PE(36:2)	+	4575	0.000	9697	0.000	0.000	<0.001	<0.001	<0.001	<0.001
PE(36:3)	-	0.000	0.000	2617	0.000	23232	<0.001	<0.001	<0.001	<0.001
PE(36:4)	+	11714	0.000	13751	0.000	0.000	<0.001	<0.001	<0.001	<0.001
PE(38:6)	-	2547	0.000	5732	0.000	0.000	<0.001	<0.001	<0.001	ns
PE(38:5)	-	6734	0.000	5851	0.000	0.000	<0.001	<0.001	<0.001	ns
PE(38:4)	-	24856	0.000	14418	0.000	0.000	<0.001	<0.001	<0.001	ns
PE(38:2)	+	2597	0.000	7455	266048	0.000	<0.001	<0.001	<0.001	<0.001
PE(40:4)	+	0.000	0.000	11536	0.000	0.000	ns	<0.001	<0.001	ns
PE(34:0(CH2))	+	15214	35923	9618	0.000	0.000	<0.001	<0.001	<0.001	<0.001
PE(44:10(OH))	-	0.000	2773	0.000	2330	0.000	<0.001	<0.001	<0.001	<0.001
PE(44:9(OH))	-	0.000	4656	0.000	2330	0.000	<0.001	<0.001	<0.001	<0.001
LPC(16:1)	+	0.000	4616	0.000	5377	0.000	<0.001	<0.001	<0.001	<0.001
LPC(16:0)	+	18841	4616	8692	5377	7647	<0.001	<0.001	<0.001	<0.001
LPC(18:2)	+	3861	35390	18841	4616	18841	<0.001	<0.001	<0.001	<0.001
LPC(18:1)	+	986.7	31479	986.7	31479	2664	<0.001	<0.001	<0.001	<0.001
LPC(18:0)	+	15366	187504	7399	151606	10504	<0.001	<0.001	<0.001	<0.001
LPC(20:4)	+	7049	0.000	39714	5527	0.000	<0.001	<0.001	<0.001	<0.001
PC(34:2)	+	0.000	0.000	12030	0.000	0.000	<0.001	<0.001	<0.001	<0.001
PC(34:4)	+	1910	0.000	7768	3482	0.000	<0.001	<0.001	<0.001	<0.001
PC(34:2)	+	200785	0.000	461328	0.000	0.000	<0.001	<0.001	<0.001	<0.001
PC(34:1)	+	191030	0.000	179388	0.000	1738	<0.001	<0.001	<0.001	<0.001
PC(36:2)	+	117896	0.000	254702	35028	0.000	<0.001	<0.001	<0.001	<0.001
PC(38:4)	+	264530	0.000	411929	0.000	0.000	<0.001	<0.001	<0.001	<0.001
PC(40:8)	+	0.000	9511	0.000	11310	0.000	<0.001	<0.001	<0.001	<0.001
LPC(18:2(OH))	+	0.000	3241	15031	4260	0.000	<0.001	<0.001	<0.001	<0.001
LPC(20:4(OH)	+	0.000	3657	47405	5527	0.000	<0.001	<0.001	<0.001	<0.001
PC(25:0(COOH))	+	4267	14295	0.000	36881	0.000	<0.001	<0.001	<0.001	<0.001
PC(34:2(OH))	+	0.000	17205	0.000	31331	1738	<0.001	<0.001	<0.001	<0.001
PC(36:4(OH))	+	439050	16217	0.000	26043	0.000	<0.001	<0.001	<0.001	<0.001
PC(36:3(OH))	+	0.000	14655	0.000	35028	0.000	<0.001	<0.001	<0.001	<0.001
PC(36:2(OH))	+	0.000	7235	0.000	21394	0.000	<0.001	<0.001	<0.001	<0.001
PC(38:4(OH))	+	0.000	8147	0.000	0.000	0.000	<0.001	<0.001	<0.001	<0.001
LPS(20:0)	-	0.000	2064	0.000	0.000	0.000	<0.001	<0.001	ns	<0.001
PS(36:4)	-	5036	0.000	1936	0.000	0.000	<0.001	<0.001	<0.001	<0.001
PS(36:2)	-	3698	0.000	2155	0.000	0.000	<0.001	<0.001	<0.001	<0.001
PS(38:4)	-	24856	0.000	14418	0.000	0.000	<0.001	<0.001	<0.001	<0.001
PS(40:6)	-	0.000	0.000	4317	550.8	0.000	ns	<0.001	<0.001	<0.001
PS(34:0)-Me	-	0.000	1082	0.000	2284	0.000	<0.001	<0.001	<0.001	<0.001
PS(38:4)-Me	-	0.000	2066	0.000	3126	0.000	<0.001	<0.001	<0.001	<0.001
LPI((16:0)	-	0.000	22761	0.000	10239	1850	<0.001	<0.001	<0.001	<0.001
LPI(18:1)	-	0.000	3894	0.000	10239	0.000	<0.001	<0.001	<0.001	<0.001
LPI(18:0)	-	1900	97652	1082	10239	23232	<0.001	<0.001	<0.001	<0.001
PI(36:4)	-	6808	3104	10013	3065	0.000	<0.001	<0.001	<0.001	<0.001
PI(38:4)	-	49327	25104	59462	18567	3092	<0.001	<0.001	<0.001	<0.001
Sphingosine (d16:1-P)	-	0.000	1990	0.000	1817	0.000	<0.001	<0.001	<0.001	<0.001

Ceramide (d42:1)	+	4371	0.000	0.000	36881	0.000	<0.001	<0.001	<0.001	<0.001
SM(d34:1)	+	65823	2373	18846	0.000	0.000	<0.001	<0.001	<0.001	<0.001
Cortisol	-	0.000	2121	0.000	1388	0.000	<0.001	<0.001	ns	<0.001
Dihydrocortisol	-	0.000	3560	0.000	3424	0.000	<0.001	<0.001	<0.001	<0.001
Cholesterol sulfate	-	46542	64394	4659	1920	0.000	<0.001	<0.001	<0.001	<0.001
Taurodeoxy-cholic acid	-	0.000	3605	3710	2485	0.000	<0.001	<0.001	<0.001	<0.001
Taurocholic acid	-	0.000	3417	10605	2233	0.000	<0.001	<0.001	<0.001	<0.001
Hydroxy-cholecalciferol	+	0.000	18286	0.000	14418	0.000	<0.001	<0.001	<0.001	<0.001
CE(20:4)	+	3893	0.000	2569	0.000	0.000	<0.001	<0.001	<0.001	ns
MG(18:1)	+	11166	0.000	69541	0.000	0.000	<0.001	<0.001	<0.001	<0.001
MG(18:0)	+	31310	3196	23976	2801	0.000	<0.001	<0.001	<0.001	<0.001
DG(32:0)	+	25206	3657	4254	0.000	0.000	<0.001	<0.001	<0.001	ns
DG(34:2)	+	25206	0.000	4254	0.000	0.000	<0.001	<0.001	<0.001	ns
DG(34:1)	+	13657	0.000	11047	0.000	0.000	<0.001	<0.001	<0.001	<0.001
DG(36:4)	+	6643	0.000	6227	0.000	0.000	<0.001	<0.001	<0.001	<0.001
DG(36:3)	+	5526	0.000	12291	0.000	0.000	<0.001	<0.001	<0.001	<0.001
DG(38:4)	+	6654	0.000	12291	0.000	0.000	<0.001	<0.001	<0.001	<0.001

* Median abundances determined from all pixel for each treatment group

** Statistical significance determined using Kruskal-Wallis followed by Dunn's test for multiple comparisons, $\alpha = 0.05$, *n* = Frozen Kidney 29587, Formalin-Fixed Kidney 22650, Frozen Liver 36374, Formalin-Fixed Liver 55233 and FFPE Liver 35543 considered pixel (Positive ion mode)/ Frozen Kidney 32300, Formalin-Fixed Kidney 25517, Frozen Liver 37022, Formalin-Fixed Liver 60958 and FFPE Liver 38569 considered pixel (Negative ion mode), ns = not significant

Figure S1:

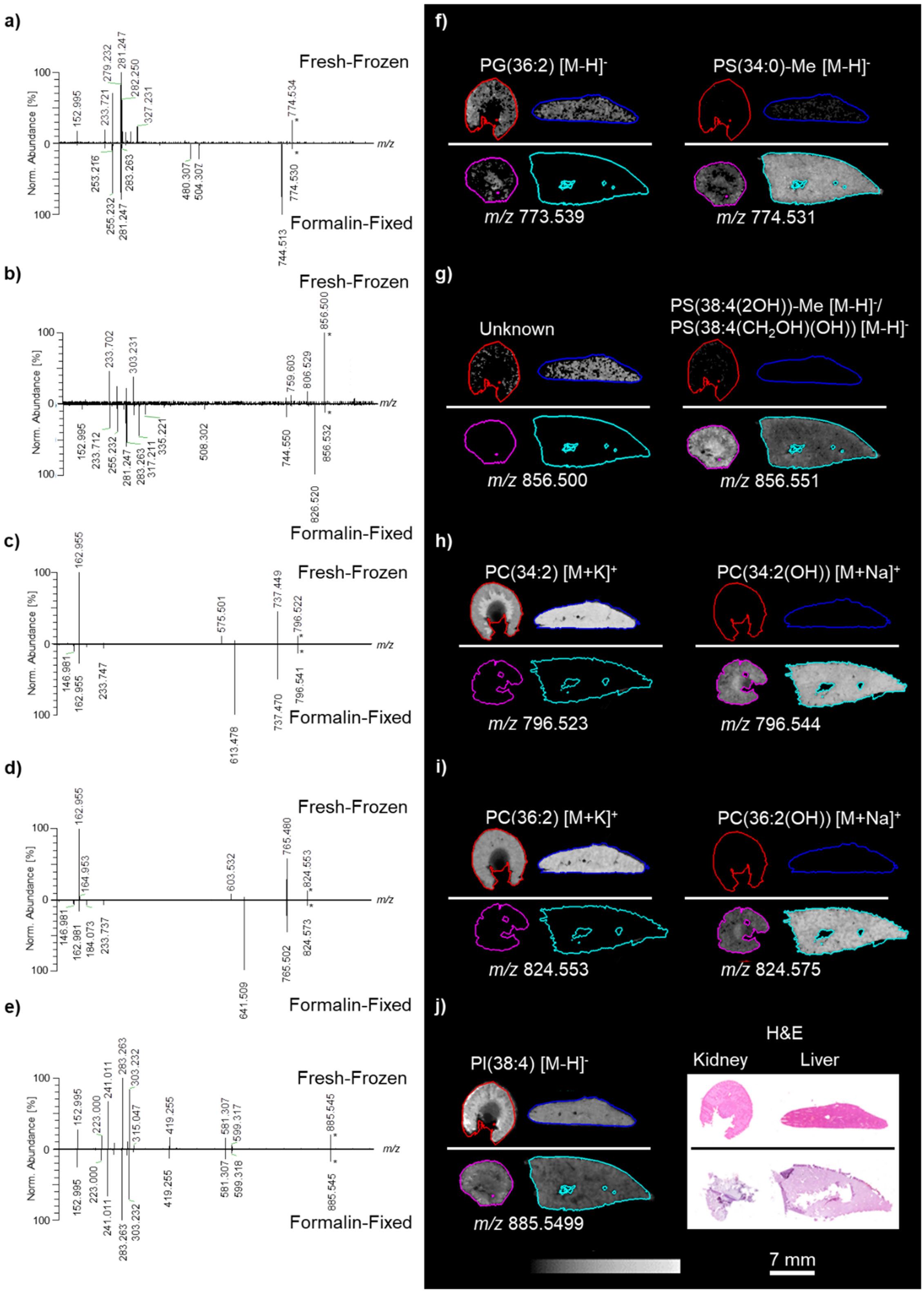

Figure S1: MS/MS spectra of heatmap lipids: in fresh-frozen (top) and formalin fixed liver (bottom) for a) m/z 774.5 (negative ion mode), 856.5 (negative ion mode), 796.5 (positive ion mode), 824 (positive ion mode) and 885.5. The * labeles the respective precursor ions in each spectrum. *a) m/z* 774.534 was found to correspond to the M+1 isotope of PG(36:2) detected as $[M-H]^-$, identifiable through the strong abundance of isotopes of the fatty acids *e.g. m/z* 282.250 as M+1 isotope of *m/z* 281.247 corresponding to FA(18:1). Isolation of the same

precursor mass on formalin-fixed liver lead to the identification of PS(34:0)-methyl ester. The methylation was elucidated from the neutral loss of 30 Da (*m/z* 774.530 to 744.513), which corresponds with the elimination of formaldehyde through comproportionation of the methanol and the carboxylic acid into the corresponding aldehydes. b) Isolation of *m/z* 856.500 from fresh-frozen tissue results in an MS/MS spectrum that could not be elucidated. The neutral loss of 50 Da (from *m/z* 856.500 to 806.529) indicates loss of CH_3Cl, which is indicative for phosphatidylcholines ionized as $[M+Cl]^-$, however, no reasonable structure could be elucidated matching the fatty acid chain composition. Isolated from formalin-fixed liver the MS/MS spectrum is dominated by the neutral loss of 30 Da indicating as above the formation of a methyl ester. The oxidation of the poly unsaturated FA(20:4), detected as shift of 2*16 Da from *m/z* 303.231 to *m/z* 335.221 for the double oxidized fatty acid and *m/z* 317.211 for the corresponding to the $[M-H_2O-H]^-$ of FA(20:4(2OH)). The oxidation complicates the determination of the exact molecular species as the spectrum does not allow to exclude the possibility of the oxidized and N-formylated PS(38:4(CH_2OH)(OH)). c/d). With the exception of MS/MS spectra obtained from PIs, the MS/MS spectra acquired from formalin-fixed samples showed overall more complexity than the MS/MS spectra for the same masses obtained from fresh-frozen specimens. e). Both precursor isolated from fresh-frozen tissue showed a neutral loss of 59 Da corresponding to the neutral loss of trimethylamine and a fragment ion detected at *m/z* 162.955 corresponding to ethylene-cyclophosphane as $[M+K]^+$ identifying the precursor as c) PC(34:2) and d) PC(36:2) detected as $[M+K]^+$. The MS/MS spectra acquired from formalin-fixed tissue share the same fragments as those from fresh-frozen tissue, but show an additional fragment at *m/z* 146.981 corresponding to the above described ethylene-cyclophosphane fragment detected as $[M+Na]^+$. The most reasonable explanation for the presence of the fragment is a shift from c) PC(34:2) and d) PC(36:2) detected as $[M+K]^+$ to PC(34:2(OH)) and PC(36:2(OH)) detected as $[M+Na]^+$ respectively. f-j) display the annotation of the deconvoluted spectra and their respective distributions in fresh-frozen (top) and formalin-fixed (bottom kidney (left) and liver (right).

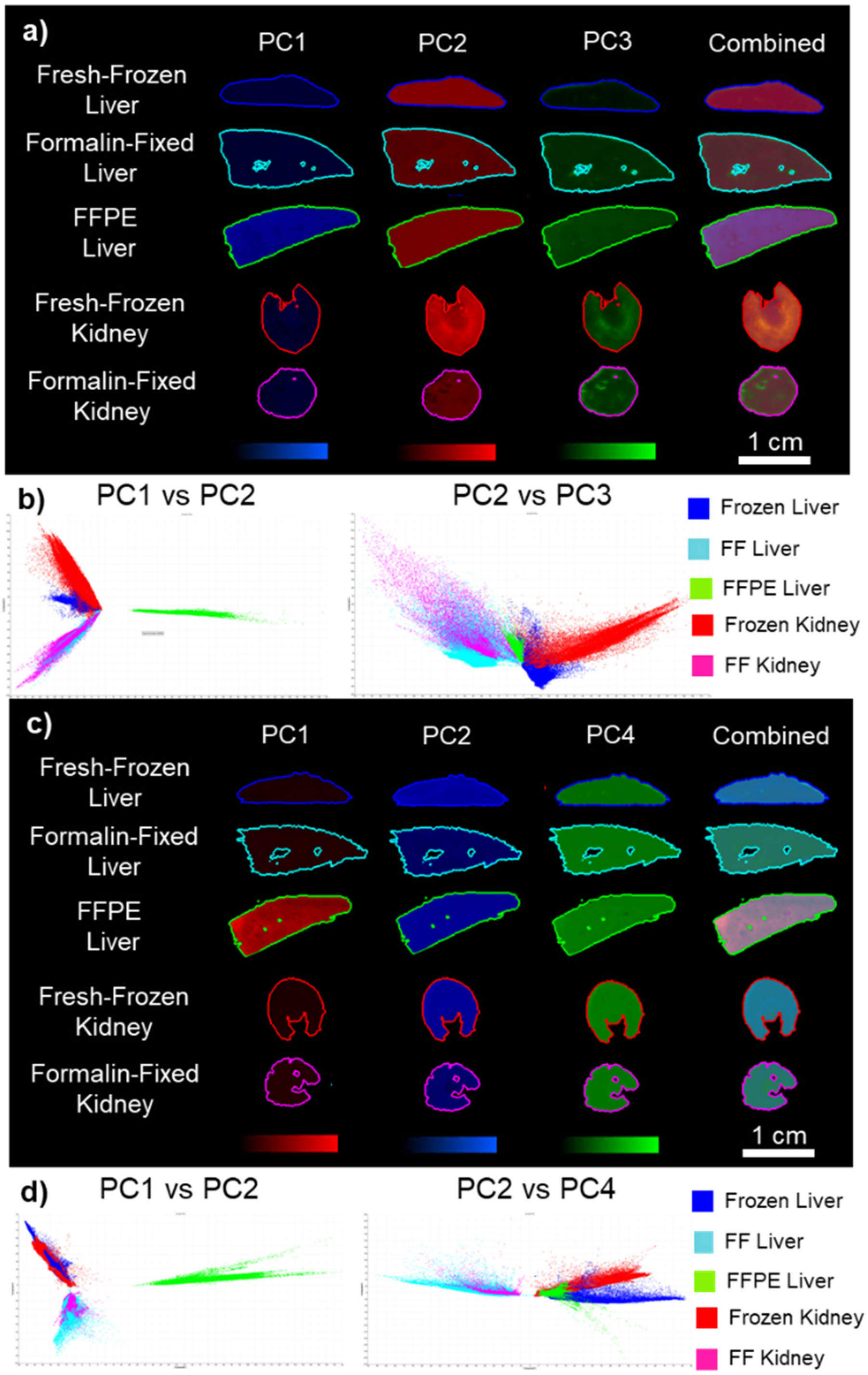

Figure S2: Pixel-wise PCA performed on sections from all tissue sections. a) shows the representative spatial PCA maps for fresh-frozen, formalin-fixed (FF) and FFPE liver as well as fresh-frozen and formalin-fixed kidneys analyzed in positive ion mode. b) Corresponding scoring plots for all samples, the left-hand side shows PC1 vs PC2 and the right-hand side PC2 vs PC3, respectively. Each datapoint represents a single spectrum. c) Representative spatial PCA maps for fresh-frozen, formalin-fixed and FFPE liver as well as fresh-frozen and formalin-fixed kidneys analyzed in negative ion mode. d) Corresponding scoring plots for all samples, the left-hand side shows PC1 vs PC2 and the right-hand side PC2 vs PC3, respectively. All displayed tissue sections originate from the same animal.

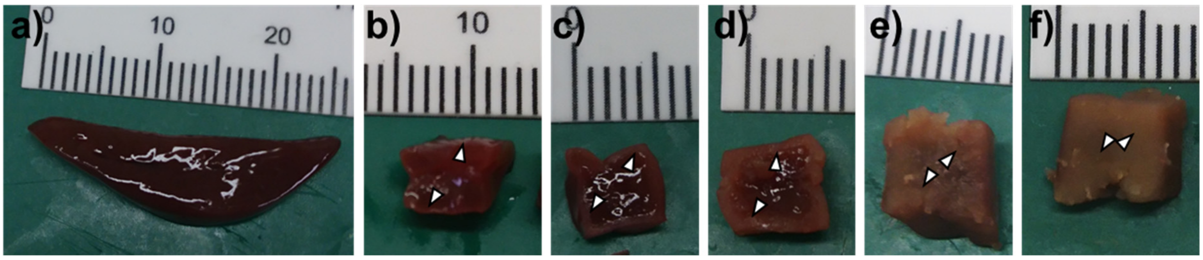

Figure S3: Progression of the fixation front in rat over time: a) Rat liver specimens of approximately 25 x 5 x 5 mm before fixation. The specimen was cut to expose the fixation front after b) 15 min, c) 30 min, d) 60 min, e) 120 min and f) 240 min in 4% formaldehyde solution. Arrowheads indicate the edge of the fixation front at the appropriate timepoints.

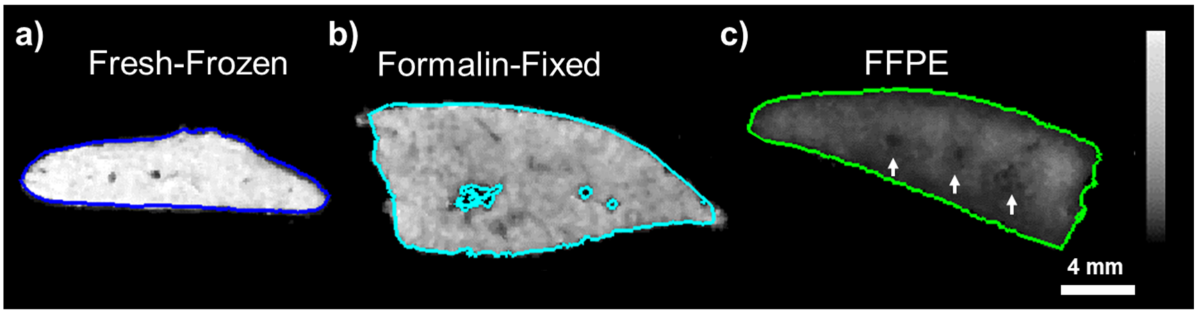

Figure S4: Distribution of PI(38:4) in a) fresh-frozen, b) formalin-fixed and c) formalin-fixed, paraffin embedded rat liver of the same animal. Arrows indicate areas of altered abundance of the lipid around larger blood vessel in the FFPE specimen.

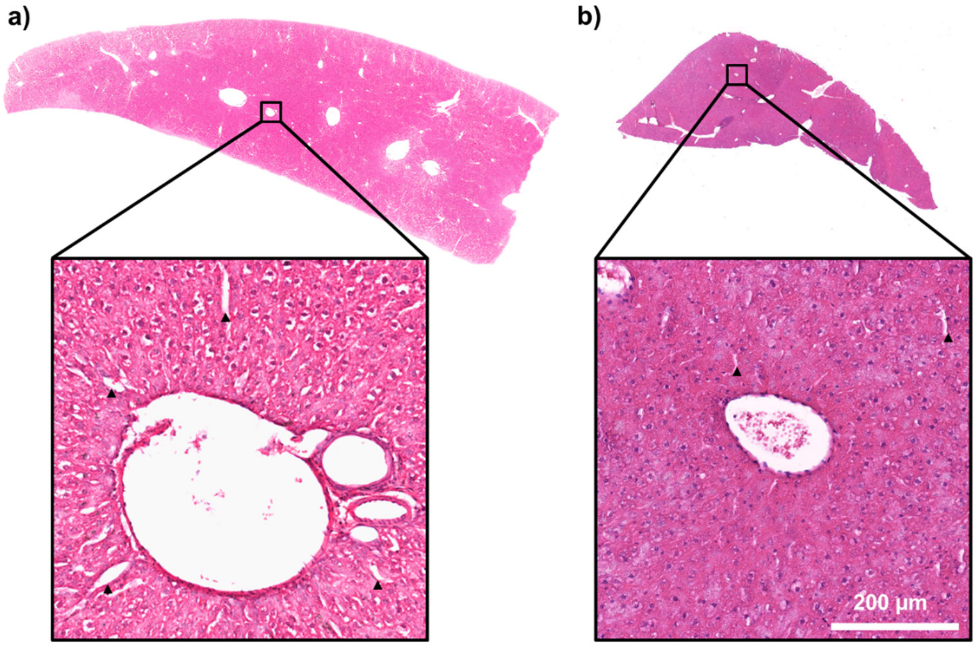

Figure S5: H&E stained FFPE sections prepared from tissue a) collected and stored in 10% formalin or b) snap-frozen upon collection and subsequently fixed in 10% formalin for 24 h before paraffin embedding. Sections were cut at 10 µm thickness, analogue to the sections analysed by MSI. The arrowheads indicate processing artefacts within the tissue sections.
